# Comparison of mitral annulus geometry between patients with ischemic and non-ischemic functional mitral regurgitation: implications for transcatheter mitral valve implantation

**DOI:** 10.1186/s12947-018-0145-8

**Published:** 2018-10-12

**Authors:** Patrizia Aruta, Denisa Muraru, Andrada Camelia Guta, Sorina Mihaila, Niccolò Ruozi, Chiara Palermo, Basma Elnagar, Sabino Iliceto, Luigi P. Badano

**Affiliations:** 1 Department of Cardiac, Thoracic and Vascular Science, University of Padua, Via Giustiniani 2, 35128 Padua, Italy; 2 University of Medicine and Pharmacy “Carol Davila”, Bucharest, Romania; 3 Tanta University Hospital, Tanta, Egypt

**Keywords:** Mitral valve, Transcatheter procedure planning, Functional mitral regurgitation, Transcatheter mitral valve replacement, 3D echocardiography, Mitral valve prosthesis

## Abstract

**Background:**

Transcatheter mitral valve replacement (TMVR) is a new therapeutic option for high surgical risk patients with mitral regurgitation (MR). Mitral valve (MV) geometry quantification is of paramount importance for success of the procedure and transthoracic 3D echocardiography represents a useful screening tool. Accordingly, we sought to asses MV geometry in patients with functional MR (FMR) that would potentially benefit of TMVR, focusing on the comparison of mitral annulus (MA) geometry between patients with ischemic (IMR) and non ischemic mitral regurgitation (nIMR).

**Methods:**

We retrospectively selected 94 patients with severe FMR: 41 (43,6%) with IMR and 53 (56,4%) with nIMR. 3D MA analysis was performed on dedicated transthoracic 3D data sets using a new, commercially-available software package in two moments of the cardiac cycle (early-diastole and mid-systole). We measured MA dimension and geometry parameters, left atrial and left ventricular volumes.

**Results:**

Maximum (MA area 10.7 ± 2.5 cm^2^ vs 11.6 ± 2.7 cm^2^, *p* > 0.05) and the best fit plane MA area (9.9 ± 2.3 cm^2^ vs 10.7 ± 2.5 cm^2^, *p* > 0.05, respectively) were similar between IMR and nIMR. nIMR patients showed larger mid-systolic 3D area (9.8 ± 2.3 cm^2^ vs 10.8 ± 2.7 cm^2^, *p* < 0.05) and perimeter (11.2 ± 1.3 cm vs 11.8 ± 1.5 cm, *p* < 0.05) with longer and larger leaflets, and wider aorto-mitral angle (135 ± 10° vs 141 ± 11°, *p* < 0.05). Conversely, the area of MA at the best fit plane did not differ between IMR and nIMR patients (9 ± 1.1 cm^2^ vs 9.9 ± 1.5 cm^2^, *p* > 0.05).

**Conclusions:**

Patients with ischemic and non-ischemic etiology of FMR have similar maximum dimension, yet systolic differences between the two groups should be taken into account to tailor prosthesis’s selection.

**Trial registration:**

N.A.

## Background

In Europe, mitral regurgitation (MR) represents the second most frequent heart valve disease after aortic valve stenosis [1]. Among patients with moderate and severe MR, 30% are affected by functional MR (FMR) with high prevalence of the ischemic etiology [2]. Despite clinical indication, 49% of patients with MR are denied for surgery due to advanced age, reduced ejection fraction or multiple comorbidities [3] and, among them, the vast majority is represented by patients with FMR [4]. In the last decade, percutaneous transcatheter procedures, simulating surgical techniques, have been developed to extend the therapeutic options for high surgical risk patients with MR. Among them, transcatheter mitral valve replacement (TMVR) represents the newest option [5–7].

Mitral valve (MV) geometry quantification is of paramount importance for the success of TMVR, and transthoracic (TTE) three-dimensional echocardiography (3DE) represents a useful tool to select the patients with the highest likelihood of uncomplicated implant [8]. It has been previously reported that MV geometry may differ in ischemic and non-ischemic FMR. In patients with ischemic MR (IMR), regional wall motion abnormalities and left ventricular [9] remodeling are more often associated with mitral annulus (MA) asymmetric dilatation [10]. Conversely, in non-ischemic MR (nIMR) global LV remodeling leads to symmetric MA dilatation [11]. Yet, MV geometry in FMR has been mainly compared to organic MR, and only few small echocardiographic studies analyzed MV geometry differentiating between IMR and nIMR [10–13]. However, none of them provided MA geometry characterization framed to pre-procedural screening for TMVR [8].

The aim of this study was to asses MV geometry in patients with FMR that would potentially benefit of TMVR, focusing on the comparison of MA geometry between IMR and nIMR patients in two key moments of the cardiac cycle —mid-systole and early-diastole.

## Methods

### Study population

Using the electronical database of the echocardiography laboratory of the department of cardiac, thoracic and vascular sciences of the University of Padua, 94 patients with severe FMR and complete transthoracic echocardiography performed between November 2010 and March 2018, have been retrospectively selected. Inclusion criteria were: age > 18 years; severe FMR according to current guidelines [14]; availability of good quality 3D data sets of both the left ventricle (LV) and the MV. We excluded patients with organic MR, mitral stenosis, aortic stenosis, more than moderate aortic regurgitation, or those with valve prostheses. Each patient was assigned to the IMR or nIMR subgroup according to his/her clinical history and the documentation of presence/absence of significant coronary artery diseases. The study was approved by the University of Padua Ethics Committee (protocol no. 70299).

### Mitral valve analysis software package validation

Two sub-studies were carried on to validate the software package used to quantitate MV geometry (4D Auto MVQ, GE Vingmed Ultrasound AS, Horten, Norway). First, the same operator (P.A.) performed the quantitative analysis of the MV in a blinded fashion, and after a time interval of one month form each other, using the same TTE data sets and both the new and a previously validated [15, 16] (4D MV Analysis; Tomtec Imaging Systems, Unterschleissheim, Germany) software packages. Second, 3D TTE and transesophageal (TEE) echocardiographic MV data sets were analyzed using the same software package for MV quantitative analysis (4D Auto MVQ, GE Vingmed Ultrasound AS, Horten, Norway) by the same operator (P.A.) in a blinded fashion, after a time interval of one week.

### Echocardiography and quantitative image analysis

**Fig. 1:**
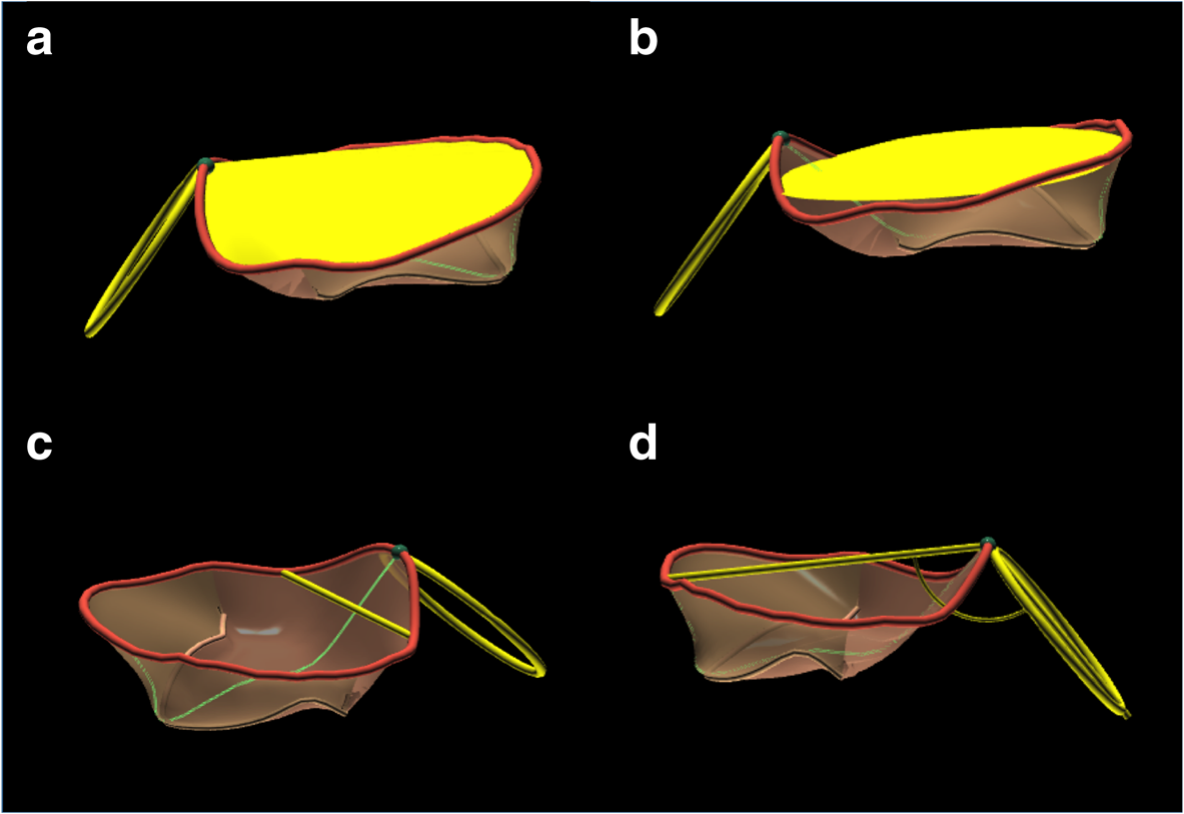
Mitral annulus parameters automatically analyzed at mid-systolic frame. Legend: Panel **a** 3D mitral annulus area, **b** mitral annulus area at the best fit plane, **c** Inter-trigonal distance, **d** Aorto-Mitral angle

All transthoracic examinations were performed using a commercially available Vivid E9 system (GE Vingmed Ultrasound AS, Horten, Norway) equipped with a 4 V probe for 3DE acquisitions according to a standardized protocol. Image analysis was performed on a dedicated workstation equipped with a commercially available software package for offline analysis of 3D datasets (EchoPac 2.02). Quantitation of LV volumes and ejection fraction (LVEF) was performed using 4D Auto-LVQ software [17] (GE Vingmed Ultrasound AS, Horten, Norway). Left atrium (LA) maximum volume was measured using the biplane disk summation method, at LV end-systole [18]. MR severity and conventional MV geometry parameters —antero-posterior (AP) and commissural (CC) diameters, tenting height and tenting area— were assessed according to current recommendations [14]. 3D MA analysis was performed on dedicated datasets by a single experienced observer (P.A.), using a new, commercially available, software package (4D Auto MVQ, GE Vingmed Ultrasound AS, Horten, Norway), in two moments of the cardiac cycle: early-diastole and mid-systole. Firstly, two time points were identified in the way that the selected frame of the analysis was midway among them. For mid-systolic analysis, the two time-points were early-systole (the frame after MV closure) and end-systole (the frame before MV begins to open). For early diastolic analysis, after identification of early-diastolic frame (first frame when MV start to open), the two time-points were placed 8 frames before and after the selected early-diastolic frame. The two orthogonal planes were adjusted to visualize the commissural and longitudinal view of MV (the longitudinal plane intersected the MV at the level of A2 and P2 scallops). For initialization, anatomic landmarks have to be added at the level of MA in the longitudinal view (posterior, P; anterior, A; leaflets coaptation point, Coap; and aortic valve, Ao) and commissural view (MA1 and MA2). The software package automatically created a 3D model of the MV in the selected frame which could eventually be edited manually, if needed (Fig. 1). Automatic quantitative parameters of the MV geometry were: MA 3D area; MA 2D area (projected 2D area at the level of the best fit plane); MA perimeter; MA AP diameter, as the distance between the two landmarks A and P; MA anterolateral-posteromedial diameter (ALPM), as the longest diameter of MA perpendicular to AP diameter; sphericity index (as the ratio between AP and ALPM diameters); MA CC diameter, as the distance between the two commissure; MA inter-trigonal distance, measured between the two automatically identified trigons; MA height, as the distance between the lowest and the highest points of MA; the non-planimetry angle, that assesses the saddle shape of MA; mitral-aortic angle, as the angle between the aortic valve and the MA (along the AP direction) planes; anterior and posterior leaflets area and length, MV tenting height, tenting area and tenting volume.

### Statistical analysis

The normal distribution of the variables was checked using the Kolmogorov-Smirnov test. Continuous data were presented as mean ± standard deviation (SD) or Median (25°-75°) and categorical variables as absolute numbers and percentages, as appropriate. In the validation study, we used Pearson or Spearman correlation to test the relationships between TTE mid-systolic MA parameters, measured using the two software packages, and mid-systolic and early-diastolic parameters obtained from TTE and TEE data sets in the same patient. In addition, Bland–Altman plots were used to assess the mean difference and the limits of agreement between them. Paired t test or Wilcoxon rank test were used, as appropriate, for comparing the MV dimension obtained by TTE and TEE data set in the same patient.

Variables were compared between IMR and nIMR patients using the unpaired t or the Mann-Whitney tests, as appropriate. Chi-square was used to compare the categorical variables. A paired t test or Wilcoxon signed rank test was used to compare systolic and diastolic dimensions within the same subgroups, as appropriate. Percentage change of the systo-diastolic measurements was also calculated.

Data analyses was performed using SPSS version 20.0 (SPSS, Inc., Chicago, IL) and GraphPad Prism V 7 (GraphPad Software, La Jolla, NY). Differences among variables were considered significant at *p* value < 0.05.

## Results

### Validation study

**Fig. 2:**
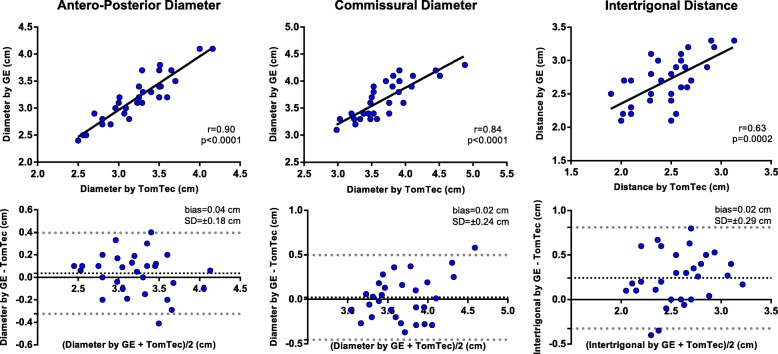
Comparisons of mitral annulus diameter measured by GE and TomTec software using Pearson correlation (top) and Bland–Altman (bottom) analyses

**Fig. 3:**
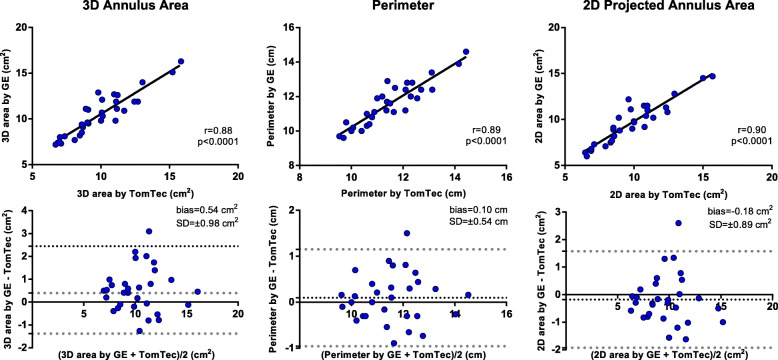
Comparisons of mitral annulus area and perimeter measured by GE and TomTec software using Pearson correlation (top) and Bland–Altman (bottom) analyses

The TTE validation cohort included 30 patients (15 with IMR; 22 men; mean age 64 ± 2 year) with good image quality. The temporal resolution of the 3D dataset for MV quantification was 35 ± 3 volumes per second (vps). Close correlations and good agreements were found between the measurements obtained with the two software packages (Figs. 2 and 3).

The TEE validation cohort included 15 patients (8 with IMR; 14 men; mean age 63 ± 15 year). As expected, both image quality (excellent quality in 75% versus 25%, respectively, *p* = 0.009) and temporal resolution (34 ± 15 vps versus 29 ± 10 vps, respectively, *p* < 0.05) were higher for TEE than TTE data sets. The mean time lapse between TTE and TEE data set acquisitions was 1(0–6) day.

Measurements obtained from TEE data sets resulted in slightly larger area, perimeter and AP diameter (Table 1). However, there was a close correlation between the two techniques and the differences were not clinically relevant. Among linear dimension, ALPM, commissural diameter and diastolic inter-trigonal distance are the most similar in TEE and TTE data sets, while tenting area, tenting volume and non-planar showed the largest differences (Table 1).

**Table 1:** Comparison of mitral annulus parameter among transthoracic and transoesophageal data sets

	Transthoracic *N* = 15	Transoesophageal *N* = 15	*p*	*r*
Diastolic dimension				
Annulus area (3D) (cm^2^)	11.5 ± 3.1	12.4 ± 3.2	0.031	0.879**
Annulus best fit plane (cm^2^)	10.6 ± 3.0	11.7 ± 3.1	0.016	0.869**
Annulus perimeter (cm)	12.1 ± 1.7	12.6 ± 1.6	0.036	0.883**
AP diameter (cm)	3.5 ± 0.5	3.8 ± 0.5	0.012	0.799**
ALPM diameter (cm)	3.7 ± 0.5	3.8 ± 0.5	0.342	0.840**
Commissural diameter (cm)	3.7 ± 0.4	3.8 ± 0.5	0.231	0.777**
Itertrigonal distance (cm)	2.8 ± 0.3	3.0 ± 0.4	0.094	0.715**
Sphericity index	0.9 ± 0.1	1.0 ± 0.1	0.150	0.157
Annulus height (mm)	7.3 ± 1.7	6.8 ± 1.6	0.343	0.559*
Non planar angle	156 ± 13	152 ± 11	0.314	0.474
Mitro-aortic angle	131 ± 8	125 ± 10	0.039	0.418
Systolic dimension				
Annulus area (3D) (cm^2^)	10.2 ± 2.6	11.0 ± 2.6	0.006	0.936**
Annulus best fit plane (cm^2^)	9.3 ± 2.5	10.2 ± 2.5	0.001	0.959**
Annulus perimeter (cm)	11.4 ± 1.5	11.8 ± 1.4	0.010	0.943**
AP diameter (cm)	3.2 ± 0.4	3.4 ± 0.6	0.002	0.624* ^ρ^
ALPM diameter (cm)	3.6 ± 0.5	3.6 ± 0.4	0.329	0.857**
Commissural diameter (cm)	3.5 ± 0.4	3.6 ± 0.4	0.151	0.889**
Itertrigonal distance (cm)	2.6 ± 0.4	3.0 ± 0.4	0.000	0.830**
Sphericity index	0.9 ± 0.1	0.9 ± 0.1	0.062	0.321^ρ^
Annulus height (mm)	7.0 ± 1.3	7.0 ± 1.4	0.910	0.300
Non planar angle (°)	153 ± 10	153 ± 9	0.893	0.240
Aorto-mitral angle (°)	139 ± 10	129 ± 8	0.005	0.367
Tenting height (mm)	10.3 ± 2.0	7.2 ± 3.6	0.02	- 0.108
Tenting area (cm^2^)	2.3 ± 0.6	2.3 ± 0.7	0.934	0.542*
Tenting volume (mL)	4.2 ± 1.3	4.1 ± 1.5	0.742	0.747**

Data are expressed as Mean ± Standard Deviation

*Abbreviations*: *ALPM* anterolateral-posteromedial, *AP* antero-posterior diameter

* for correlation with *p* < 0.05; ** for correlation with *p* < 0.001; ^ρ^ evaluated with Sperman’s correlation

### Comparison of mitral annulus geometry between ischemic and non-ischemic mitral regurgitation

We enrolled 94 patients, 41 (43,6%) with IMR and 53 (56,4%) with nIMR. Patients with IMR were more frequently male and had a higher incidence of hypertension, diabetes and dyslipidemia (Table 2). The severity of MR was comparable between the two groups (Table 3). Although patients in both groups showed severe LV dilatation and dysfunction, patients with IMR had a higher LVEF (31 (26–38)% versus 28 (22–32)%, *p* = 0.030) and LV wall motion score index (2.1 ± 0.3 versus 1.9 ± 0.6, *p* = 0.021) (Table 3).

**Table 2:** Demographics and clinical characteristics

	Ischemic mitral regurgitation *N* = 41	Non ischemic mitral regurgitation *N* = 53	*p*
Age (years)	69 (63–75)	64 (55–72)	0.081
Men (%)	*35 (85)*	*35 (66)*	*0.033*
Body surface area (m^2^)	1.8 (1.7–1.9)	1.9 (1.7–2.0)	0.374
Heart rate (bpm)	71 (59–85)	75 (65–86)	0.237
Systolic blood pressure (mmHg)	110 (100–120)	100 (95–115)	0.340
Diastolic blood pressure(mmHg)	65 (60–71)	65 (60–70)	0.233
Hypertension	*32 (80%)*	*25 (48.1%)*	*0.002*
Diabetes	*15 (37.5%)*	*8 (15.4%)*	*0.015*
Dyslipidemia	*30 (75%)*	*22 (42.3%)*	*0.002*
Smokers	23 (57.5%)	24 (46.2%)	0.280
Resynchronization therapy	8 (20%)	17 (32.7%)	0.175

Data are expressed as Median (25°-75°) or Number (%). Italicized values highlight statistically significant differences

**Table 3:** Echocardiography characteristics

	Ischemic mitral regurgitation *N* = 41	Non ischemic mitral regurgitation *N* = 53	*p*
MR Vena contracta (mm)	7 (6–8)	7 (6–8)	0.658
MR PISA radius (mm)	7 (6–8)	8 (7–9)	0.138
MR EROA (mm2)	2 (2–3)	2.1(2–3)	0.421
MR R Vol (mL)	38 (28–58)	38.5 (29–47.7)	0.803
sPAP	47 (35–56)	44 (35–49)	0.211
TR severity	Trivial 6 (11.3%)	Trivial 6 (15.4%)	0.753
	Mild 29 (54.7%)	Mild 20 (51.3%)	
	Moderate11 (20.8%)	Moderate10 (25.6%)	
	Severe 7 (13.2%)	Severe3 (7.7%)	
AR severity	None 25 (49%)	None 25 (65%)	0.437
	Trivial 13 (25%)	Trivial 6 (15%)	
	Mild 12 (23.5%)	Mild 9 (22.5%)	
	Moderate 1 (2%)	Moderate 0 (0%)	
LV EDV (ml/m^2^)	134 (114–153)	143 (116–178)	0.078
LV ESV (mL/m^2^)	96 (68–109)	105 (78–135)	0.075
Ejection Fraction (%)	*31 (26–38)*	*28 (22–32)*	*0.030*
Indexed LA volume (mL/m2)	*60 (51–68)*	*70 (53–91)*	*0.031*

Data are expressed as Median (25°-75°) or Number (%)

*Abbreviations*: *AR* aortic regurgitation, *EROA* effective regurgitant orifice area, *LA* left atrial, *LV EDV* left ventricular end-diastolic volume, *LV ESV* left ventricular end-systolic volume, *MR* mitral regurgitation, *PISA* proximal isovelocity surface area, *R Vol* regurgitant volume, *sPAP* systolic pulmonary artery pressure, *TR* tricuspid regurgitation. Italicized values highlight statistically significant differences

Temporal resolution of the 3D dataset dedicated for MV quantification was higher in IMR than in nIMR patients (33 ± 14 vps versus 40 ± 16 vps, *p* = 0.023). All data sets had enough good quality for the quantitative analysis. The image quality was graded excellent in 47 patients (50%), good in 32 (34%), and fair in 15 (16%) and it was comparable between IMR and nIMR patients (*p* = 0.634).

Using conventional two-dimensional echocardiography MV geometry parameters, patients with nIMR showed larger AP diameter both in diastole (41 ± 7 mm in nIMR versus 38 ± 6 mm in IMR, *p* = 0.029) and in systole (37 ± 6 mm in nIMR versus 34 ± 4 mm in IMR, *p* = 0.024). Conversely, CC diameter (43 ± 8 mm in nIMR versus 39 ± 9 mm in IMR, *p* = 0.088), tenting height (9 ± 3 mm in nIMR versus 8.5 ± 3 mm in IMR, *p* = 0.180) and tenting area (1.9 ± 0.7 cm^2^ in nIMR versus 1.7 ± 0.6 cm^2^ in IMR, *p* = 0.189) were similar between the two groups.

At 3DE analysis, both subgroups had similar diastolic geometry of MA, even though all MA dimensions were slightly larger in nIMR. nIMR patients showed larger mid-systolic 3D area and perimeter of the MA with longer leaflets. However, the area of the annulus at the best fit plane, and all diameters (AP, CC, ALPM diameter and trans-trigonal distance) did not differ between IMR and nIMR patients. Tenting height and area did not differ between IMR and nIMR patients, whereas tenting volume, annulus height and aorto-mitral angle were larger in nIMR patients (Table 4).

**Table 4:** Three-dimensional mitral valve dimension

	Ischemic mitral regurgitation *N* = 41	Non ischemic mitral regurgitation *N* = 53	*p*
Diastolic dimension			
Annulus area (3D) (cm^2^)	10.7 ± 2.5^a^	11.6 ± 2.7^a^	0.124
Annulus best fit plane (cm^2^)	9.9 ± 2.3^a^	10.7 ± 2.5^a^	0.135
Annulus perimeter (cm)	11.7 ± 1.4^a^	12.2 ± 1.4^a^	0.111
AP diameter (cm)	3.3 ± 0.4^a^	3.5 ± 0.5^a^	0.072
ALPM diameter (cm)	3.6 ± 0.4^a^	3.8 ± 0.5^a^	0.129
Commissural diameter(cm)	3.6 ± 0.4	3.7 ± 0.4	0.300
Itertrigonal distance (cm)	2.7 ± 0.4^a^	2.8 ± 0.3^a^	0.374
Annulus height (mm)	6.3 ± 1.7^a^	6.8 ± 1.7^a^	0.144
Sphericity index	0.9 ± 0.1^a^	0.9 ± 0.1^a^	0.963
Non planar angle (°)	156 ± 11^a^	153 ± 10^a^	0.232
Anterior leaflet area (cm^2^)	7.5 ± 1.6^a^	8.0 ± 1.6^a^	0.142
Posterior leaflet area (cm^2^)	7.2 ± 2.3^a^	7.5 ± 2.1^a^	0.413
Anterior leaflet length (cm)	2.9 ± 0.4	3.3 ± 0.9^a^	0.102
Posterior leaflet length (cm)	1.6 ± 0.4^a^	1.7 ± 0.6^a^	0.319
Aorto-mitral angle (°)	131 ± 9^a^	135 ± 11^a^	0.115
Systolic dimension			
Annulus area (3D) (cm^2^)	*9.8 ± 2.3*	*10.8 ± 2.7*	*0.046*
Annulus best fit plane (cm^2^)	9 ± 2.1	9.9 ± 2.5	0.063
Annulus perimeter (cm)	*11.2 ± 1.3*	*11.8 ± 1.5*	*0.048*
AP diameter (cm)	3.1 ± 0.4	3.2 ± 0.5	0.063
ALPM diameter (cm)	3.5 ± 0.4	3.7 ± 0.5	0.065
Commissural diameter(cm)	3.5 ± 0.4	3.7 ± 0.4	0.130
Itertrigonal distance (cm)	2.5 ± 0.3	2.7 ± 0.3	0.051
Annulus height (mm)	*6.7 ± 1.6*	*7.5 ± 1.9*	*0.047*
Sphericity index	0.9 ± 0.08	0.9 ± 0.1	0.598
Non planar angle (°)	153 ± 11	150 ± 10	0.268
Anterior leaflet area (cm^2^)	*6.5 ± 1.6*	*7.4 ± 1.7*	*0.006*
Posterior leaflet area (cm^2^)	*5.7 ± 1.7*	*6.5 ± 1.9*	*0.049*
Anterior leaflet length (cm)	*2.8 ± 0.6*	*3 ± 0.4*	*0.022*
Posterior leaflet length (cm)	*1.3 ± 0.4*	*1.5 ± 0.8*	*0.022*
Aorto-mitral angle (°)	*135 ± 10*	*141 ± 11*	*0.011*
Tenting height (mm)	9.3 ± 2.6	10.3 ± 2.8	0.082
Tenting area (cm^2^)	2.2 ± 0.7	2.4 ± 0.8	0.141
Tenting volume (mL)	*4 ± 1.7*	*4.7 ± 1.7*	*0.047*

Data are expressed as Mean ± Standard Deviation

*Abbreviations*: *ALPM* anterolateral-posteromedial, *AP* antero.posterior diameter

Italicized values highlight statistically significant differences

^a^Statistical difference vs systolic dimension

### Mitral annulus dynamics

In both groups, MA significantly reduced its dimensions in systole (except for the CC diameter) with similar percentage change of the measurement in both groups (*p* > 0.05) (Tables 4 and 5). During systole, the MA mitral-aortic angle flattens, while the non-planarity angle becomes more acute.

**Table 5:** Fractional changes of the mitral annulus parameters between diastole and systole

	Ischemic mitral regurgitation *N* = 41	Non ischemic mitral regurgitation *N* = 53	*p*
MA area (3D) fraction (%)	-6 (-11.7 — -1.8)	-4.3 ( -9.8 — -1.3)	> 0.05
MA best fit plane fraction (%)	-6.3 (-13 — -4)	-6.7 (-11.4 — -1.8)	> 0.05
MA perimeter fraction (%)	-3.2 (-5.8 — -0.4)	-2.1 (-4.6 — -0.8)	> 0.05
AP diameter fraction (%)	-7.4 (-11.4 — -2.4)	-5.9 (-11.2 — -1.3)	> 0.05
ALPM diameter fraction (%)	-2.9 (-6.9 — 0.0)	0.0 (-7.3 — 0.0)	> 0.05
CC diameter fraction (%)	-2.6 (-5.5 — 2.9)	0.0 (-4.7 — 2.7)	> 0.05
TT distance fraction (%)	-4.8 (-12.4 — 0.0)	-3.1 (-10 — 3.8)	> 0.05
Non planar angle fraction (%)	-2 (-6.3 — 2.5)	-2.4 (-5.6 — 2)	> 0.05
Aorto-mitral angle fraction (%)	3.7 (-2.5 — 8.4)	3.4 (0–8.4)	> 0.05

Data are expressed as Median (25°-75°)

*Abbreviations*: *ALPM* anterolateral-posteromedial, *AP* antero-posterior diameter, *CC* commissural, *MA* mitral annulus, *TT*, trans-trigonal

## Discussion

In the present study, we used 3D TTE to compare MA geometry in patients with severe ischemic and non-ischemic FMR, who are potential candidates for TMVR.

The main findings of our study were in patients with FMR: i, diastolic MA geometry is similar in both nIMR and IMR patients; ii, systolic MV geometry significantly differs between the groups.

### Validation study

Multimodality imaging represents the gold standard for planning transcatheter mitral valve procedures, TEE and multi-slice computed tomography (MSCT) playing the major role [19]. Due to longer survival of patients with chronic heart diseases and progressive aging of the general population, the number of patients who could benefit of TMVR is likely to increase, and 3DTTE will be of paramount importance as a screening tool for the analysis of MV geometry. Previous clinical studies assessing MA geometry used 3DTEE data sets [20, 21] to obtain adequate spatial and temporal resolution for quantitative analysis of the MV. However, the progressive improvement of 3DE technology allows to obtain better and better quality 3DE data sets with TTE, too. Moreover, feasibility and cost/effectiveness considerations suggest that TTE approach would be better suited to screen potential candidates to TMVR. Accordingly, we decided to explore the use of 3DTTE data sets perform quantitative analysis of the MV in patients with FMR. In our patients, MA dimensions obtained from TTE datasets were similar to those obtained with the 3D TEE approach in the validation study.

### Comparison of mitral annulus dimension between ischemic and non-ischemic mitral regurgitation

We focused our study on patients with FMR because they represent the main potential target of new TMVR. The few previous studies that analyzed the possible differences between IMR and nIMR [10–13] included a limited number of patients and were focused only on MA size (annulus area and diameters), without any information about the MV geometry (MA area at the best fit plane, mitro-aortic angle, length of the anterior leaflet) which are crucial to select patients for TMVR [19].

In this study, we reported all MV anatomical and geometrical features that should be assessed before TMVR [8, 22] and demonstrated that patients with severe IMR and nIMR have similar, symmetrical, diastolic (maximal) MA dimension. The 3D MA area obtained from our patients were comparable with the maximum MA surface area reported by Veronesi et al. [12] in a smaller group of patients using TTE 3DE datasets. Our results are also in agreement with those reported by Daimon et al. [10] who showed that diastolic MA diameters did not differ among IMR and nIMR. However, the actual MA sizes in our patients were slightly larger than in their cohorts. This finding could be partially explained by the different time point selected for the analysis (mid-diastolic phase, compared to early-diastole in our study).

While in our study mid-systolic 3D annulus area and perimeter are significantly larger in patients with nIMR, MA area at the best fit plane and MA diameter were similar. It has been suggested that the projected 3D MA area at the level of the best fit plane is the most reliable parameter of MA geometry to be used for planning TMVR compared to the saddle-shaped 3D area [22]. Though, our MA area at the best fit plane resulted smaller than the mean projected MA area measured in a recent MSCT study [23] on 32 patients with FMR of different etiologies and severity, it is already known that 3DE can underestimate measurement compared with MSCT due to its suboptimal lateral resolution in the coronal plane [24].

A new D-shaped MA segmentation developed by Blanke et al. [25], with the truncation of anterior saddle horn at the level of inter-trigonal line, has been used to select candidates to Tiara [5], Tendyne [7] and Intrepid [6] valve implants. This method was also recently applied by Mak et al. [26] using 3D TEE with comparable results, but it is unclear at this early stage of TMVR experience whether this is the best parameter to size the prosthesis for TMVR interventions [27].

Left ventricular out flow tract (LVOT) obstruction is a possible complication related to TMVR that can be predicted during procedure planning because it is related to the design of the prosthesis and patient anatomy (interventricular septal dimension, LV size, aorto-mitral angle, anterior leaflet length). 3DE allows the measurement of both the aorto-mitral angle (the angle between the aortic valve and the MA along the AP direction) and anterior leaflet length. None of the previous MSCT nor the 3DE studies reported these parameters in patients considered for TMVR. In our study we found that nIMR group presented significantly wider aorto-mitral angle that balance the potential higher risk of LVOT obstruction due to longer and larger anterior leaflets in these patients.

### Mitral annulus dynamics

MA is dynamic structure characterized by contraction and expansion phase during cardiac cycle [12, 28]. These changes, although less pronounced than in normal subjects, have been reported also in patients with IMR [20, 29] and nIMR [12]. We found that in patients with severe FMR, MA is significantly smaller in mid-systole compared to early diastolic phase. This findings underline the necessity of a multiphasic MA assessment to select patients for TMVR [8], but the few investigations that analyzed MA dimension in moderate or severe FMR (potentially candidates for TMVR), reported only the measurement in one phase of the cardiac cycle [12, 13].

### Implications for trans-catheter mitral valve selection

TMVR represents a promising option for patients with severe FMR, and assessment of MA dimension and geometry is of paramount importance to size the device and also to plan future development of new prostheses. We found that, patients with IMR and nIMR have similar MA geometry, supporting the concept that there is no need of different prosthesis sizing according to etiology of the FMR. However, we found that nIMR patients had significantly larger and longer anterior mitral leaflet, that could increase the risk of LVOT obstruction. Therefore, for nIMR patients it could be more appropriate to select a device that has an anterior hook to fix the anterior leaflet of the native MV. On the other hand, nIMR patients showed a wider aorto-mitral angle that could counterbalance the higher risk of LVOT obstruction carried by longer anterior leaflet. Probably, this sub-group of patients would be eligible also for devices that have larger protrusion or flaring into LV.

The significant change of MA during the cardiac cycle, also preserved in patients with severe FMR, stresses the need to evaluate accurately the smallest MA dimension in order to reduce the risk of excessive stress of the prosthesis frame by MA.

### Study limitations

We acknowledge several limitations of our study. First, to obtain all the measurements needed to plan TMVR from 3DE data sets, we used a new MV software package that was not previously validated. To overcome this limitation, we compared the measurements obtained with the new software package with those obtained from the same data sets using a validated software [16] with a close correlations and good agreement. However, we did not compare our measurement with MSCT, which represents the current gold standard to select patients for TMVR.

Secondly, currently available 3DE software packages allow MV dynamic analysis only during the systolic phase of the cardiac cycle. While mid-systole could be defined by the operator according to MV opening and closure or automatically by the software (as mid-way between R and T waves on the ECG tracing), early-diastole has to be manually identified by the operator with an increased possibility of errors. Current literature reports contradicting data about the moment when MA reaches its maximum and minimum sizes, however the importance of definition of maximum MA dimension is of paramount importance for accurate device’s sizing and emphasizes the need of multiphasic annular measurement.

## Conclusion

The reported MA geometry in a relatively large group of patients with severe FMR, potentially candidates for TMVR, represents useful information for transcatheter MV prosthesis design and patient selection. Patients with ischemic and non-ischemic aetiologies of FMR have similar maximum dimensions, yet systolic differences between the two groups should be taken into account to tailor prosthesis’s selection.

## Abbreviations

3DE: Three-dimensional echocardiography
ALPM: Anterolateral-posteromedial
AP: Antero-posterior
CC: Commissural
FMR: Functional mitral regurgitation
IMR: Ischemic MR
LA: Left atrium
LV: Left ventricle/ventricular
LVEF: Ejection fraction
LVOT: Left ventricular outflow tract
MA: Mitral annulus
MR: Mitral regurgitation
MSCT: Multi-slice computed tomography
MV: Mitral valve
nIMR: Non-ischemic mitral regurgitation
TMVR: Transcatheter mitral valve replacement
TEE: Transesophageal echocardiography
TTE: Transthoracic echocardiography
VPS: Volumes per second
